# Automatic Evaluation Algorithms for Radio Tomography Imaging Methods

**DOI:** 10.3390/s25061747

**Published:** 2025-03-12

**Authors:** Krzysztof Strzecha, Grzegorz Rybak

**Affiliations:** Institute of Applied Computer Science, Lodz University of Technology, ul. Stefanowskiego 18, 90-537 Lodz, Poland; grzegorz.rybak@p.lodz.pl

**Keywords:** RTI evaluation, FEM, image reconstruction, LBP, people detection, simulation, radio tomography

## Abstract

The radio tomography imaging (RTI) method is very similar to X-ray tomography, but it operates in the radio frequency band without exposing the human body to harmful tissue-penetrating radiation. It can be used to monitor the number of people and their locations in buildings such as offices or hospitals. RTI can be useful in emergencies, rescue operations, and security breaches. The novelty of this paper includes the flexible architecture of an evaluation platform for RTI image reconstruction algorithms, as well as an automated evaluation process. The concept of the developed platform assumes the use of a distributed architecture based on microservices. Numerous advantages of the proposed architecture are pointed out. The presented approach ensures flexibility for further development work thanks to the system’s high degree of granularity and modularity.

## 1. Introduction

Industrial process tomography is a non-invasive imaging technique used in a variety of applications, giving the opportunity to increase the efficiency of process visualization, control, and management. It plays an important role in continuous measurement, allowing for a better understanding and monitoring of industrial processes. It provides quick and dynamic responses that facilitate real-time industrial process control, error detection, and system failure recognition. There are plenty of tomographic techniques used in the industry. The most commonly used is electrical tomography, including resistive and capacitive tomography. Ultrasound, X-ray, or gamma-ray tomography are used as well. These types of techniques can also be successfully used in the implementation of object identification systems, in particular in people detection systems in buildings [1]. The human body disrupts radio waves, making it possible to estimate its position and track its movements [2]. In this paper, the authors discuss radio tomography imaging (RTI) for people detection, as it is a common method of Device-Free Localization (DFL) and has been a rapidly developing technology in recent years [3].

RTI can be useful in emergencies, rescue operations, and security breaches. The use of radio tomography images to track people moving inside closed spaces is the basis for new applications in security systems and so-called “smart buildings” [4,5]. The RTI method is very similar to X-ray tomography but operates in the radio frequency (RF) band, such as the Industrial, Scientific, Medical Band (ISM), without exposing the human body to harmful tissue-penetrating radiation [6,7,8]. ISM is a radio band initially intended for industrial, scientific, and medical applications. An important feature that distinguishes the RTI technique from other solutions is its wave propagation characteristics. Unlike other types of transmission tomography, ultrasound tomography and radio tomography have one feature in common: ultrasound and radio waves propagate in straight lines [9].

Recent studies have used so-called received signal strength (RSS) measurements on 802.11 WiFi to detect and locate people [2] or 5G mobile systems [3], although these techniques involve problems such as signal interference or expensive deployment [4]. The simulations presented in [10] demonstrate the capabilities of a detector based on signal strength measurements in determining the location of a person who is not wearing an additional electronic device. The system is trained by people in predefined locations, and RSS measurements are recorded at each location. When the system is in use, the RSS measurements are compared with known training data and the best position is selected [6].

Radio tomography systems can be divided into two groups. The first group includes static systems, while the second one is based on dynamic solutions, including mobile ones. One of the mobile tomography solutions is presented in [11]. The transmitting antenna and the receiving antenna are placed on two mobile platforms outside the building, enabling the acquisition of information about its internal structure. The transceiver platform scans the space in different directions, according to the principle of computed tomography (CT). Directional antennas are used to reduce environmental influences (e.g., multipath). To guarantee a low observation error, the main plane of the transmitting antenna is always aligned with the main plane of the receiving antenna. Wireless connectivity is maintained at each location. The signal is transmitted by the transmitter and the RSS is measured at each position of the sensors. RSS measurements are obtained as a coherent set of information that is used to reconstruct Spatial Loss Field (SLF) images [11].

In RTI, each transmitter in a Wireless Sensor Network (WSN) repeatedly sends packets to all other transmitters. If an object physically stand on the signal path, the strength (RSS) will decrease in relation to the values obtained during calibration [12]. The system discussed in [6] only measures signal strength. There is no information about the phase or timing of the signal. In practice, the radio (received) Signal Strength Indicator (RSSI) expressed in dB or dBm (dB per milliwatt) is used. There are two types of RTI systems, which comprise Ultra-Wide-Band (UWB) broadband systems and Ultra-Narrow-Band (UNB) narrowband systems. In addition, two groups of tomographic methods can be distinguished [13]: reflective tomography and transmission tomography, based on the so-called “shadow”.

The use of RF tomography, as opposed to a much higher frequency (e.g., X-rays), introduces significant Non-Line-Of-Sight (NLOS) propagation into the measurements. Signals in the commercial band do not propagate only in the Line of Sight (LOS), but in many directions from the transmitter to the receiver. Despite some disadvantages, the experimental results show that RTI is able to visualize RF attenuation in dense wireless networks with inexpensive and standard equipment [6].

There are plenty of scientific articles that describe various RTI realizations. Paper [6] presents a linear model of using Received Signal Power (RSP) measurements to obtain images of moving objects. Computed tomography (CT) methods in medical and geophysical imaging systems use signal measurements along many different paths through a medium. Measurements along the paths are used to estimate the spatial field of transmission parameters in the entire medium [14]. RTI is also a transmission-based imaging method that measures signal strength in many different paths through the medium, but like radar systems, it does so at radio frequencies.

As part of a study enabling image reconstruction, a transmission model was used in [15]. The measurement system consisted of 16 antennas. The measured values were the power expressed in dBm in a straight line between the individual antennas. On the other hand, in Wilson’s research [6], a wireless peer-to-peer network with 28 nodes was implemented to test the ability of RTI to analyze the phenomenon of electromagnetic wave attenuation. This approach was possible thanks to the use of the 802.11 wireless network in the 2.4 GHz band. The main task of the presented device was to send frames in the iBeacon standard via the Bluetooth module and to scan the environment in search of devices operating in the same standard. When the device detects the so-called Beacon, it automatically reads its identification data and signal strength and sends them to the broker via the MQTT protocol. Then, these data and data from other Beacons are analyzed and processed into an image [15,16].

Another possible implementation is a battery-powered measuring device based on iBeacon, WiFi, MQTT, and Raspberry Pi technologies. In order to implement the system, tools such as message brokers and Bluetooth technologies are used [14]. In turn, the solution presented in [17] uses proprietary sets of small devices with the implementation of popular wireless standards such as Bluetooth, Wi-Fi, ZigBee, or Z Wave, which are also mentioned in [3].

As described above, the RTI technique is constantly being developed, and the possibilities of its use are increasing. Unfortunately, there are no solutions that would allow for an automatic verification of the developed systems. This study fills this research gap by proposing a flexible and modular evaluation platform that enables the automated testing and comparison of RTI reconstruction techniques. The research presented in the following sections is a response to the existing need. The novelty of the work presented in the article is the automatic evaluation process of imaging methods in radio tomography and the flexible architecture of the evaluation platform. The main contribution of this work is the RTI reconstruction evaluation algorithm, along with the concept of a highly modular system. Unlike previous solutions, the presented method attempts to automatically evaluate radio tomography image reconstruction algorithms.

## 2. Materials and Methods: RTI Techniques and Reconstruction Algorithms

Objects in the measurement area of radio tomography systems absorb, reflect, diffract, or disperse part of the transmitted power [15]. The purpose of the RTI system is to define an image vector that describes the radio power attenuation caused by physical objects placed in the region of the sensor network. Such networks consist of several transmitters, where each pair of two devices stands for a basic RTI link.

**Figure 1:**
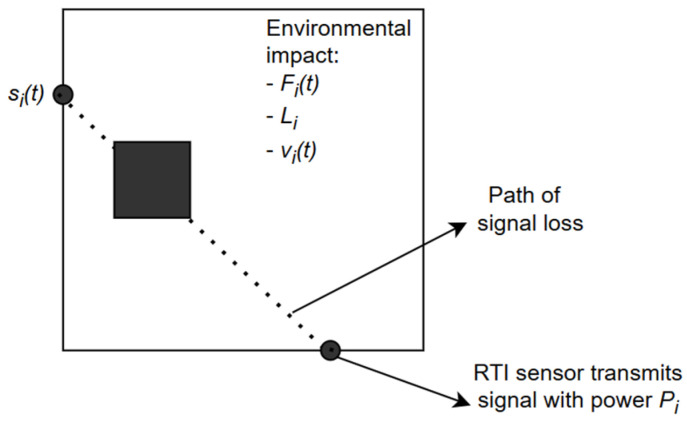
Model of a single RTI link with the presented signal loss path between two radio devices. An obstacle constituting a source of signal loss is modeled between them.

Figure 1 presents a simple RTI link where one transmitter generates a signal with strength $P_{i}$ and the other receives reduced strength $y_{i}\left(t\right)$. The reduction is primarily caused by the influence of the object crossing the link.

Let *K* be the number of RTI network nodes. Then, the total number of unique two-way connections is given by $M=\frac{K^{2}-K}{2}$. Each pair of nodes is considered a link, regardless of whether actual communication exists between them. The signal strength $y_{i}\left(t\right)$ of a specific link depends on the parameters described below [15]:

(1) $$y_{i}\left(t\right)=P_{i}-L_{i}-S_{i}\left(t\right)-F_{i}\left(t\right)-\nu_{i}\left(t\right)$$

where

$P_{i}$—transmitted power (dB);

$L_{i}$—static losses (dB) due to distance, antenna characteristics, device inconsistencies, etc.;

$S_{i}\left(t\right)$—shading loss (dB) caused by objects that attenuate the signal;

$F_{i}\left(t\right)$—the decaying loss (dB) that occurs due to the constructive and destructive interference of narrowband signals in multipath environments;

$\nu_{i}\left(t\right)$—measurement noise.

As the resulting image consists of voxels, the shading loss $S_{i}\left(t\right)$ can be approximated as the sum of the attenuation that occurs in each voxel. Since each voxel’s contribution to link attenuation is different for each link, weighting is used. For a single link, it is described by the formula

(2) $$S_{i}\left(t\right)=\sum_{j=1}^{N}w_{ij}x_{j}\left(t\right)$$

where $x_{j}\left(t\right)$—attenuation occurring in voxel j at time t; $w_{ij}$—weight of voxel j for connection i.

If the link does not “cross” a particular voxel, that voxel is removed using a weight of zero value. Only imaging the varying attenuation greatly simplifies the problem, since all static losses can be removed. The RSS change $\Delta y_{i}$ from time $t_{a}$ to $t_{b}$ is given by

(3) $$\Delta y_{i}\equiv y_{i}\left(t_{b}\right)-y_{i}\left(t_{a}\right)=S_{i}\left(t_{b}\right)-S_{i}\left(t_{a}\right)+F_{i}\left(t_{b}\right)-F_{i}\left(t_{a}\right)+\nu_{i}\left(t_{b}\right)-\nu_{i}\left(t_{a}\right)$$

which can be written as

(4) $$\Delta y_{i}=\sum_{j=1}^{N}w_{ij}\Delta x_{j}+n_{i}$$

where noise $n_{i}$ is the grouping of fading and measurement noise

(5) $$n_{i}=F_{i}\left(t_{b}\right)-F_{i}\left(t_{a}\right)+\nu_{i}\left(t_{b}\right)-\nu_{i}\left(t_{a}\right)$$

and

(6) $$\Delta x_{j}=x_{j}\left(t_{b}\right)-x_{j}\left(t_{a}\right)$$

is the attenuation difference in pixel j over time from $t_{a}$ to $t_{b}$.

Other important issues are the influence of external disturbances on the propagation of electromagnetic waves and the noise recorded during the measurements. Variation in RSS over time, when no moving object blocks the path of the radio wave links, is perceived as noise in the RTI system. The research and measurements carried out by Bultitude [18] were used to design indoor stationary wireless communication systems that periodically experienced dropouts due to movement in the measurement area. Bultitude [18] found that the RSS signal undergoes significant attenuation due to human movement within the network area. Most of the time, the measured RSS changes slowly around a nearly constant average, which is called the fading interval. During the decay period, the RSS varies up to 10 dB above and 20 dB below the average of the fading interval, with a distribution that can be characterized as a Rician distribution [18,19]. Other measurements show that the statistics of the attenuation over time follow a log-normal distribution more closely [20]. At all times, the measurements show a two-part mixture distribution of RSS for a fixed link. In linear terms, these data can be modeled as a combination of two Rician distributions [18]. They can also be modeled as a combination of log-normal distributions, as suggested by the results in [20]. The models described above regarding the factors influencing the spatial representation of the real positions of objects that are the main cause of signal attenuation can be reduced to a linear problem.

**Figure 2:**
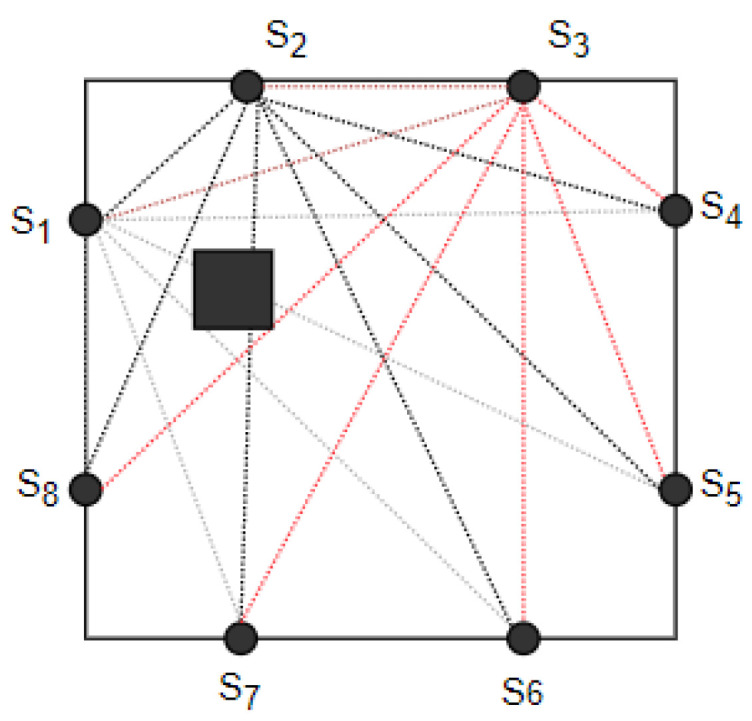
Diagram of an RTI network where all nodes transmit a signal and all nodes receive the signal and analyze its strength. The obtained information can be used to reconstruct the image and identify the location of objects in the area of the network. Example links for transmitter S3 are presented in red.

The basis for the operation of RTI systems is the use of multiple transmitters. Each sends a signal, and while transmitting, the others will measure the strength of the signal coming to them. The image is obtained by combining the information from the sensors, as well as predetermined parameters. An illustration of the RTI network is shown in Figure 2.

If all links in the network are considered simultaneously, the system of RSS equations can be described in matrix form. A linear model for RTI can take the following form [12,14,15,16,17,18,19,20,21,22,23,24]:

(7) Δy=WΔx+n

where

$$\begin{array}{l} \Delta y={\left[\Delta y_{1},\Delta y_{2},\ldots ,\Delta y_{M}\right]}^{T}\\ \Delta x={\left[\Delta x_{1},\Delta x_{2},\ldots ,\Delta x_{N}\right]}^{T}\\ \;\;\;n={\left[n_{1},n_{2},\ldots ,n_{M}\right]}^{T}\\ \;\;{\left[W\right]}_{i,j}=w_{ij} \end{array}$$

In summary, Δy is the RSS difference measurement vector of length M for all links, **n** is the noise vector, and Δx is the attenuation image to be estimated. **W** is an M × N weighting matrix where each column represents a single voxel and each row describes the weight of each voxel for that particular link. Each variable is measured in decibels (dB).

**Figure 3:**
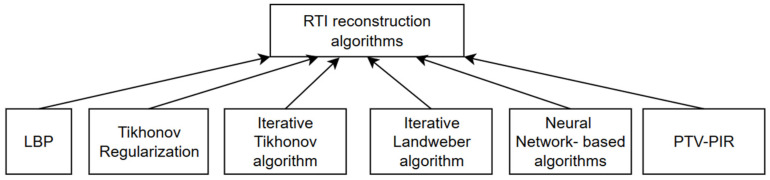
Image reconstruction algorithms.

The heuristic explanation for the ill-posed [6] RTI problem is that many pixels are estimated from a relatively small number of nodes [8,15,25]; therefore, there is a deficit of the explanatory variables in relation to the dependent variables [1]. There are many possible attenuation patterns that can lead to the same set of measurement data. For example, suppose a given pixel is not intersected by any link in the network. This would yield the same measurement data for every possible attenuation value of that pixel, so reversing the problem would be impossible. There are many image reconstruction algorithms. In Figure 3, the main algorithms of tomographic image reconstruction are presented.

The most often used algorithms are based on regularization. Regularization [26] is the introduction of additional information to an ill-posed problem in order to improve the quality of the solution. It is often used in solving inverse problems. Among the regularization methods developed, the following can be mentioned:

- Tikhonov regularization [6,13,14,15,24,25];
- Regularization by singular value decomposition (TSVD [4], SVD [9]);
- Iterative regularization methods;
- Regularization by discretization;
- Regularization by filtering.

For the purposes of their research, the authors of [11] considered various reconstruction algorithms: LBP, Tikhonov regularization, the iterative Landweber algorithm, and the total variation minimization (TV) algorithm. Their advantages and disadvantages are mentioned below. In [1], the authors use convolutional neural networks (CNNs).

The LBP method is a very simple and effective way to reconstruct an image. It is a useful tool for real-time processing. The LBP algorithm requires the least hardware resources and can be implemented even in devices or sensors that have very limited memory, but the image quality is not good enough for most applications. LBP is able to meet the requirements of specific scenarios, such as localization [8].

Tikhonov regularization gives better results compared to the LBP method. The quality of Tikhonov regularization strongly depends on the regularization parameter λ. The authors of paper [8] use the L-Curve method to obtain a reference value for λ. Unfortunately, the disadvantage of Tikhonov regularization is the need to perform an inversion, which has high computational complexity [8]. Although regularization methods can achieve stable results, the resulting images are quite blurry. In general, the reconstructed image of the wall has the so-called sparse structure characteristics in the spatial domain [11].

On the other hand, the Landweber iteration method gives the best results compared to Tikhonov regularization and the LBP algorithm, and it does not require the calculation of an inverse matrix. The method requires many iteration steps before obtaining satisfactory results, which takes a long time. It is typically used with an off-line image reconstruction approach for better imaging results.

Another algorithm considered was the total variation (TV) minimization algorithm. Its advantage compared to regularization methods is that the image reconstruction result can be clearer. The TV minimization algorithm also has disadvantages such as “staircase” effects. There may be artifacts in the final results due to measurements at a limited angle and model errors [11]. An improved TV minimization algorithm for image reconstruction called RTV-PIR is given in [11]. Interpreting the obtained image and determining the position of the object in an RTI image involves identifying the area of pixels with the highest values (local maxima). Effects such as noise and interference affect the accuracy of detection. The algorithm presented in [4] traverses all the feature region combinations and outputs the localization result, thanks to which it is possible to estimate the position of real objects more accurately.

For the purposes of this article, the authors decided to use the LBP algorithm, as it is the fastest one. As the solution presented in this paper refers to an automation process, it is important to highlight that the image reconstruction algorithm is implemented as a component and can thus be changed at any time.

## 3. The Automatic Evaluation of RTI Reconstruction Algorithms

The boundary element method helps to solve engineering problems related to differential equations. BEM is also referred to as the boundary integral equation method (BIEM) because it is effective in solving boundary value problems. The finite element method (FEM), in turn, consists in discretizing the computational domain into so-called finite elements using a local coordinate system, simple polynomial functions, and the Jacobi matrix. Both numerical methods are used in modeling of tomographic imaging [27]. The following study focuses on the application of the FEM using available numerical calculation systems software. FEM grids and electromagnetic wave modeling algorithms for the selected RTI measurement areas were prepared.

There are many tools that allow you to perform calculations using the finite element method. The most commonly used are Matlab R2024b, FreeFEM v4.15, EIDORS v3.12 (Electrical Impedance and Diffuse Optical tomography Reconstruction Software; the original EIDORS software is based on the software from Vaukhonen [28,29]), and FEniCSx v0.9, a tool described in detail in A. Logg’s publication [30].

Modeling phenomena that use so-called coupled systems to solve problems are a difficult issue. Examples of such phenomena are fluid–structure interactions or Lorentz forces. The FreeFem v4.15 program allows for the effective modeling of such phenomena using partial differential equations for non-linear systems. It allows for the modeling of phenomena using unstructured and custom 2D or 3D meshes [31]. It is possible to use different types of triangular finite elements, including discontinuous elements. The main advantages of the software are its ability to enter geometric data, which allows for the easy description of boundaries, and the generation of arbitrary meshes of different densities. The platform includes many linear direct and iterative solvers. In addition, the software allows you to generate and save many types of reports enriched with various graphics.

### 3.1. The Architectural Concept of the RTI Evaluation Platform

**Figure 4:**
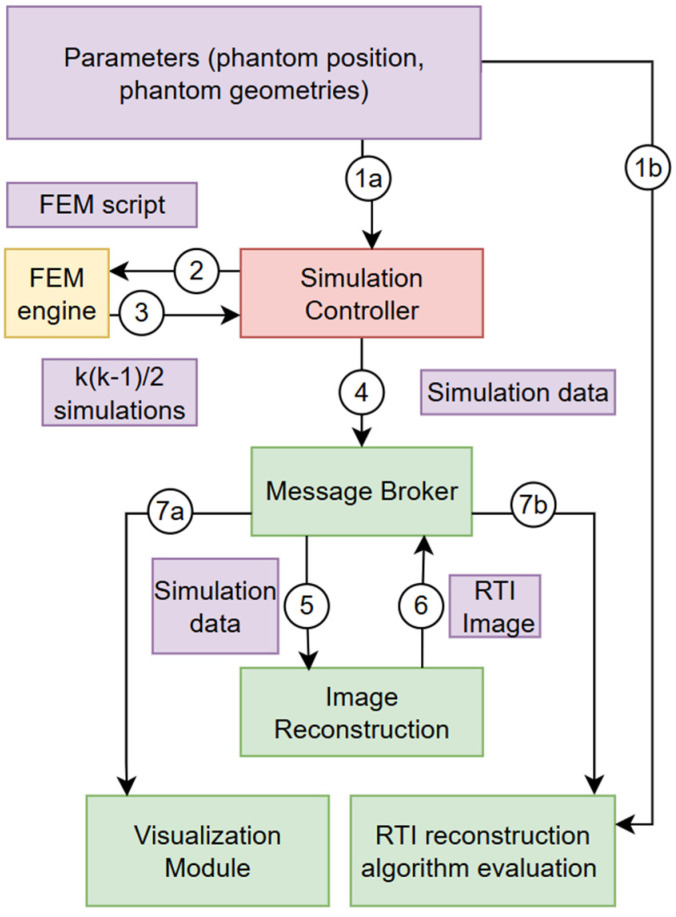
A conceptual diagram of the RTI reconstruction evaluation platform. The numbers on the arrows represent the next steps of data flow.

For the purposes of the presented research, the FreeFEM v4.15 engine was selected. Based on this tool, a platform for evaluating RTI reconstruction algorithms was prepared. First, it was necessary to develop an architectural concept for the solution. The chosen concept assumes the use of a distributed architecture based on microservices. Thanks to early functional decomposition and a high level of software granularity, it is possible to later integrate the solution with cloud components in the SaaS model. Figure 4 presents the main implementation units of the developed platform.

The concept includes a simulation controller, a FEM engine, a message broker, an image reconstruction module, a visualization module, and an analysis module. The identified objects that are the basis for communication between the developed modules are also indicated. These include simulation parameters, a FEM script, simulation results with K elements, where $K=k*\left(k-1\right)/2$ and *k* is the number of transmitters, a bundle of complete simulation data, and an RTI output reconstructed image. In Figure 4, the numbers next to the arrows indicate the successive steps of the algorithm, which are described in detail in the next section.

### 3.2. Evaluation Process

**Figure 5:**
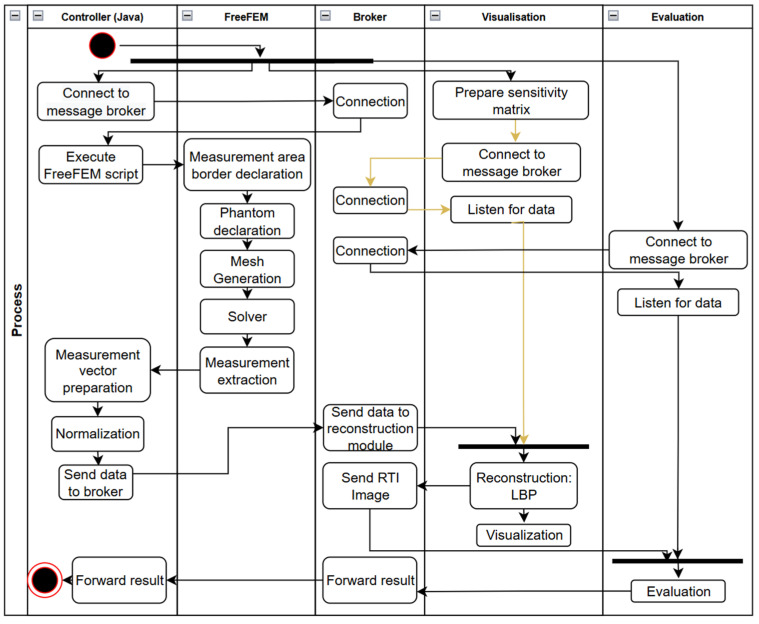
Activity diagram of RTI reconstruction evaluation algorithm with FEM simulations.

The process begins with launching the tools in the distributed environment. First, a message broker must be launched to allow subsequent modules to connect to a common communication channel. At the same time, the simulation controller is connected and the sensitivity matrix is prepared in the visualization module. The reconstruction and visualization modules are connected to the broker and then switched to listening for messages sent by the broker. An activity diagram for the automated verification process of RTI image reconstruction algorithms using FEM tools is presented in Figure 5.

The FEM engine (here, FreeFem v4.15) is started via the simulation control module. The FreeFem script contains a sequence of actions that lead to a simulation of the distribution of the RSSI parameter in a bounded area, also entered as a script parameter. The operation starts with declaring the boundaries of the measurement area. Then, information about the shape and location of the phantom in the simulated model is introduced.

The FEM mesh is generated and then the solver (an algorithm that calculates values for the FEM nodes) is run. After obtaining the simulation data in the form of a calculated parameter distribution, the extraction of the simulation value is performed only at the locations of the simulated sensors. The data are transferred to the simulation controller. This module processes the received data into a measurement vector, which is processed using a normalization algorithm. Finally, the normalized vector is sent to the broker via a previously declared communication channel. The visualization module receives a message that aggregates all the necessary information needed to perform image reconstruction. Then, reconstruction is performed and the image data are sent to the visualization module, which ends with obtaining the image on the so-called canvas. At the same time, the data go to the analysis module and to the broker. This allows for their integration with external tools, including cloud solutions.

### 3.3. Mesh Generation

**Figure 6:**
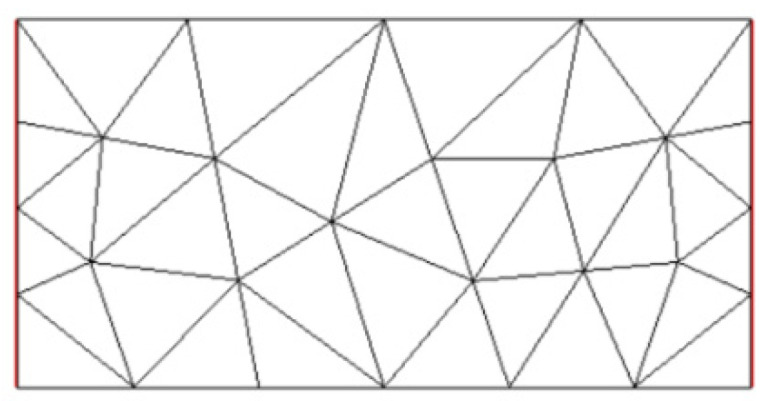
FEM meshes in shape of rectangle.

The mesh generation phase is performed in order to proceed with the finite element method. Listing 1 shows the code for generating rectangular meshes in FreeFEM v4.15. The results are shown in Figure 6.
**Listing 1.** Source code of mesh generation for rectangular area.border wallA(n=0, 2){x=n; y=0; label=1;}; border wallB(n=0, 1){x=2; y=n; label=1;}; border wallC(n=0, 2){x=2-n; y=1; label=1;}; border wallD(n=0, 1){x=0; y=1-n; label=1;}; mesh Th = buildmesh(wallA(6) + wallB(4) + wallC(4) + wallD(4)); plot(Th, wait=true, fill=true, ps=“rectangle.eps”);

### 3.4. Solver—RSSI Distribution

Since the signal duration is short, the power will not change during the measurement. Without time analysis, so-called stationary solutions are sought, and Maxwell’s equations of electromagnetic wave propagation can be simplified to a single Helmholtz equation:

(8) $$\nabla^{2}E+\frac{{vn}^{2}}{n^{2}}E=0$$

where *vn* is the angular wave number of the WiFi signal and *n* is the refractive index of the material through which the wave propagates.

The subject of this study is the influence of objects in the signal path on its power. The refractive index n for an obstacle is different from that the refractive index for air (equals 1). For walls, the refractive index is a complex number in which both parts have a physical interpretation: the real part specifies the reflection of the wall and the imaginary part specifies the absorption of the wall.

**Figure 7:**
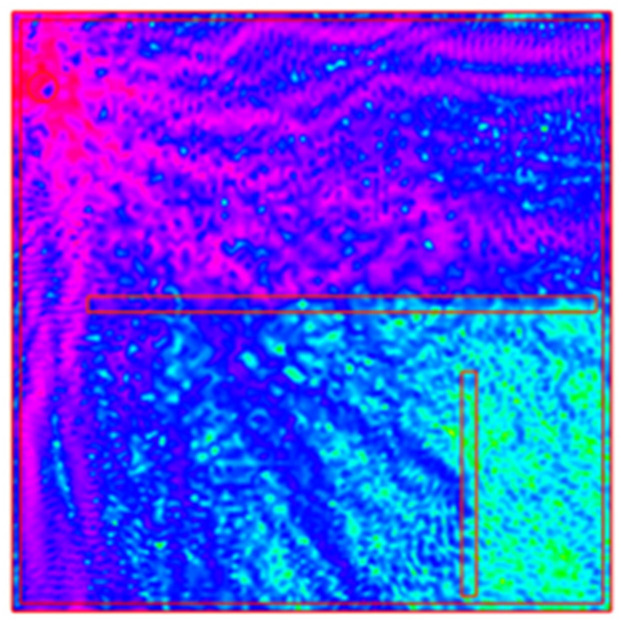
Construction of the FEM image—electromagnetic wave propagation model with the example of WI-FI systems (source: https://doc.freefem.org/tutorials/wifiPropagation.html, accessed on 9 March 2025).

To calculate the RSSI distribution in a two-dimensional space, the formula provided with the FreeFEM v4.15 tool (Listing 2) was used, and the result image is presented in Figure 7.
**Listing 2.** Solver source code.problem muwave(v,w) = int2d(Th)( (v*w*k^2)/(1+(wallreflexion+wallabsorption)*wall())^2              - (dx(v)*dx(w)+dy(v)*dy(w))) + on(2, v=1);

### 3.5. Data Extraction from FEM Simulation

Data are passed to the main program via the FreeFEM v4.15 output stream. Four types of signals are used in the form of formalized character strings, which allows for correct handling of data downloads. The indicated signals include simulation start, FEM image data, measurement vector in JSON format, and simulation end signal.

### 3.6. LBP Image Reconstruction

**Figure 8:**
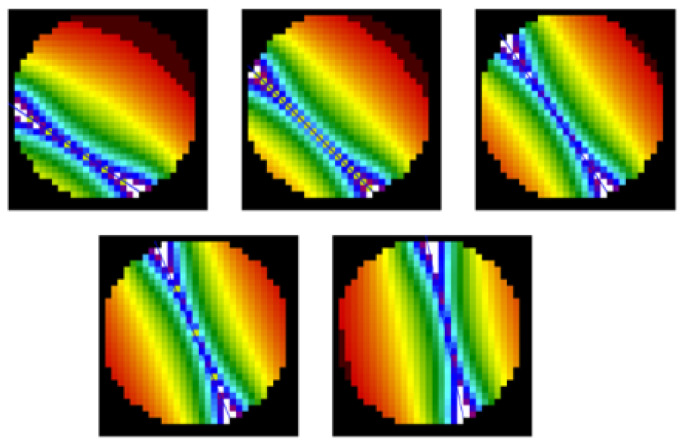
Fragment of the sensitivity matrix for 16 transmitters.

Preparation of the measurement data vector for reconstruction is performed based on the extraction of appropriate simulation values for the indicated pairs of tomographic sensor electrodes. The list of electrode pairs is presented in Listing 3. During vector preparation, the normalization procedure was also performed.
**Listing 3.** A fragment of the test controller source code, which allows for the construction of a measurement vector.ArrayList<String> SM_8_0_32x32 = Lists.*newArrayList*( “5|4”, “5|3”, “5|2”, “5|1”, “5|0”, “5|7”, “5|6” , “4|3”, “4|2”, “4|1”, “4|0”, “4|7”, “4|6” , “3|2”, “3|1”, “3|0”, “3|7”, “3|6” , “2|1”, “2|0”, “2|7”, “2|6” , “1|0”, “1|7”, “1|6” , “0|7”, “0|6” ,  “7|6”);

The measurement vector is prepared and transmitted via the WebSocket protocol to the reconstruction and visualization modules in a web browser. The RTI reconstruction code uses the LBP algorithm and previously prepared sensitivity matrices (programming language: JavaScript). A fragment of the developed sensitivity matrix is presented in Figure 8.

LBP reconstruction is performed using the code presented in Listing 4. The first step of the algorithm is to create a 2D array for the result data. Secondly, iteration through each part of the sensitivity matrix is performed, and each pixel of the output image is calculated.
**Listing 4.** A snippet of the LBP algorithm source code.var reconstructed = that.create2dArray(32,32); for(var measurement=0; measurement<28; measurement++) { for(var posX=0; posX < 32; posX++) { for(var posY=0; posY < 32; posY++) { var v = that.data[measurement][posX][posY] * measurements[measurement]; reconstructed[posX][posY]+= v || 0; }}}

**Figure 9:**
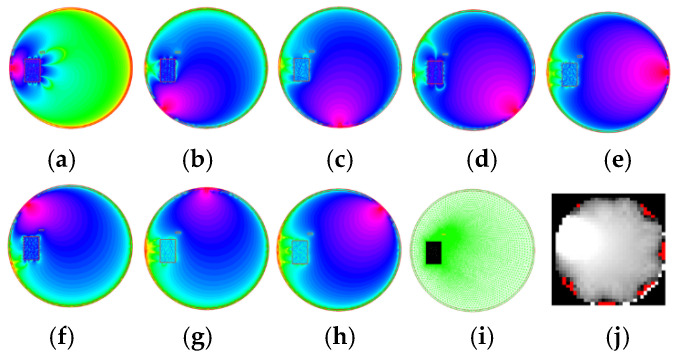
FEM simulation (**a**–**h**) with visualized mesh refinement (**i**) and reconstructed RTI image (**j**) for eight radio transmitter positions. The angular shift for the resulting image results from the sensitivity matrix applied.

Figure 9 demonstrates how FEM data are converted into an RTI image. Figure 9a–h show the 2D distribution of WIFI signal strength for every transmitter, calculated using an irregular mesh (Figure 9i). Figure 9j shows the RTI output image obtained with the LBP reconstruction algorithm. As might be seen, the simulated object, which is an obstacle in the LOSS path, is also visible in the output image. The angular shift in the result image is due to the sensitivity matrix used.

## 4. Simulations and Results

**Figure 10:**
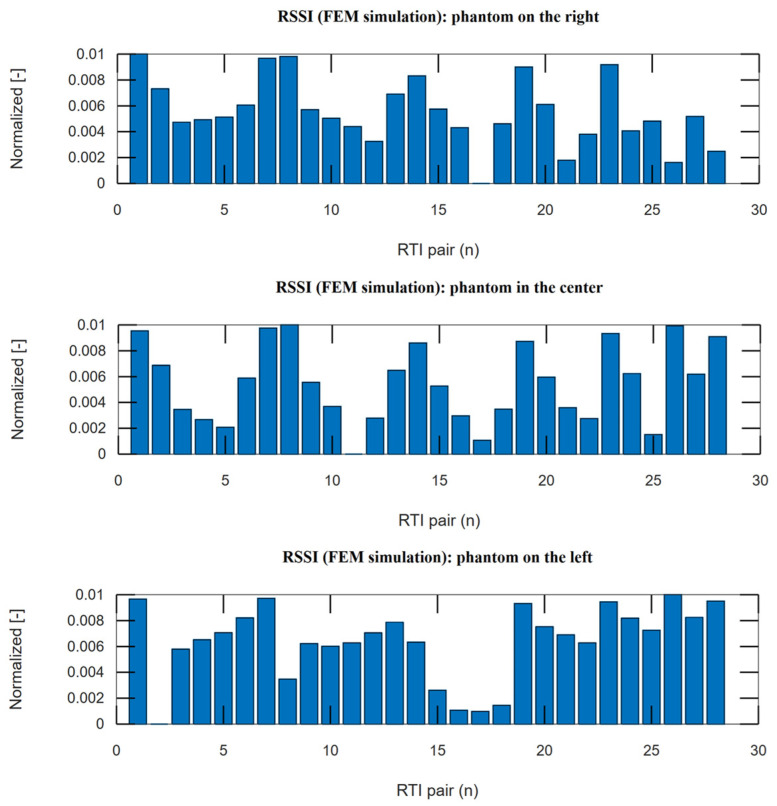
Plot of simulated RTI measurement vectors for several obstacle object locations. Location of the obstacle object in the measurement space (from **top image**): right; center; left.

A series of complex and time-consuming tests were conducted. Measurements were taken for multiple locations of the obstacle object. At least eight FEM images were generated for each location. Each image was transformed into a vector representing a set of simulated values. For each obstacle object position, a vector of 28 simulated values was obtained and then passed to the RTI reconstruction algorithm as input. Examples of simulated measurement vectors are presented in Figure 10, which consists of three obstacle object locations.

**Figure 11:**
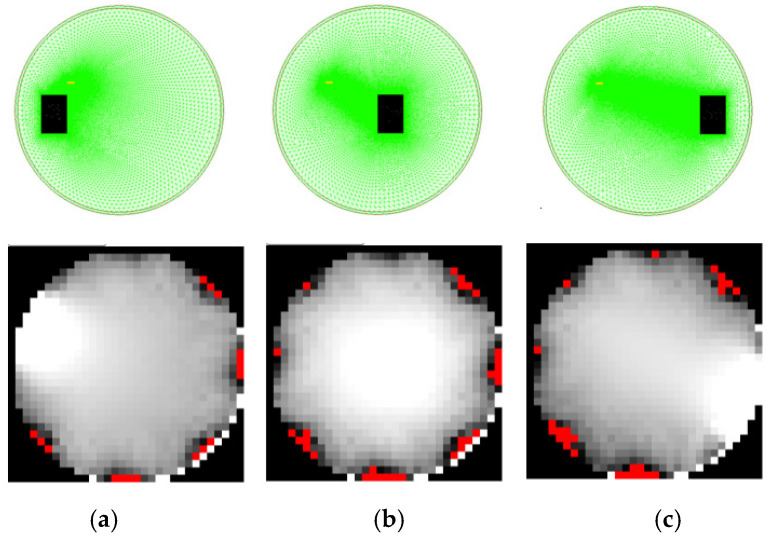
Reconstruction of RTI images based on FEM-generated vectors with a mesh model for 8 transmitters; (**a**) leftmost object position in the measurement space; (**b**) center object position; (**c**) right object position. The angular shift for the resulting image results from the sensitivity matrix applied.

The data presented in Figure 10 were obtained using automatically generated meshes. Obstacle objects were located as shown at the top of Figure 11. The figure also presents RTI simulation images, which clearly indicate where objects were detected in the measurement space.

**Figure 12:**
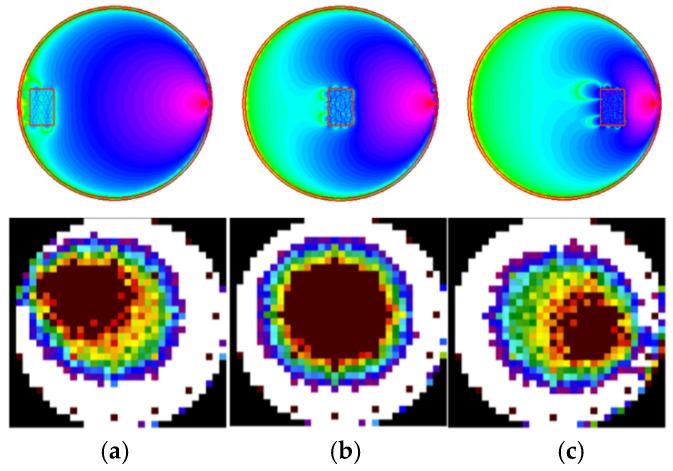
Reconstruction of RTI images based on FEM-generated vectors with a mesh model for 16 transmitters (**top row**); (**a**) leftmost object position in the measurement space; (**b**) center object position; (**c**) right object position. The angular shift in the resulting image results from the applied sensitivity matrix.

During the second simulation, the phantom object locations changed slightly. The number of transmitters was increased from eight to sixteen. Additionally, a color value scale was used to visualize the RTI image. The results are presented in Figure 12.

This study also included the analysis of algorithm execution times, both for the direct problem and the inverse problem, using the LBP algorithm. *A* PC computer with the following parameters was used for this research: processor Intel(R) Core(TM) i7-10750H CPU @ 2.60 GHz 2.59 GHz, 32.0 GB RAM, 64-bit Windows 11 operating system, Amazon Corretto 11.0.25 Java environment, and JavaScript for visualizing the reconstruction results. The algorithm execution times are presented in Table 1.

The simulation time for 8 transmitters and 16 transmitters was analyzed. The simulation time (forward problem) is the total time for all pairs of transmitters. The reconstruction time is responsible for the reconstruction of one image based on the measurement vector.

## 5. Discussion

RTI is an imaging method based on the transmission of radio waves that measures the power of a signal propagating many different paths in a medium. Radio waves propagate in straight lines. RSS is measured at each sensor position. Objects within the measurement area of an RTI system absorb, reflect, diffract, or dissipate part of the transmitted power. The purpose of the RTI system is to define an image vector describing the radio signal power attenuation caused by physical objects in N voxels of the network region.

In the case of RTI, there are environmental factors that affect the accuracy of reconstruction: multipath interference, noise variations, and object material properties. Radio signals often propagate along multiple paths due to reflections and scattering, which can result in both constructive and destructive interference. This variability in received signal strength (RSS) can introduce errors in the reconstruction process if it is not properly taken into account. Both intrinsic sensor noise and external environmental fluctuations contribute to measurement uncertainty. In addition, dielectric properties, conductivity, and other factors specific to the materials of objects in the measurement space influence signal attenuation and reflection. What is important is how these properties can alter RSS measurements and consequently affect RTI accuracy. These critical factors should also be a subject of detailed studies in the field of automatic quality assessment systems for tomographic image reconstruction techniques.

A platform for the automatic evaluation of RTI image reconstruction algorithms was designed and implemented. In terms of technical aspects, numerous advantages of the proposed architecture were pointed out. The concept of the developed platform assumes the use of a distributed architecture based on microservices. This approach ensures flexibility for further development work thanks to the system’s high degree of granularity and modularity. The components are reusable and can be dynamically replaced using interfaces and a consistent domain model. There is no need to restart the system when internal modules are changed.

The FreeFEM v4.15 tool, used in conjunction with the parameterized script developed, allowed for the simulation of the RSSI parameter’s distribution over a bounded, specified area. During the process, data were transferred to the simulation controller. Reconstruction was performed, and then the image data were sent to the visualization function. Among the many image reconstruction algorithms, the LBP method, which enables fast, real-time image reconstruction, was implemented. The conducted research allowed for the formulation of the following conclusions:

1. Automating the reconstruction algorithm verification process accelerates the development of the RTI platform;
2. Separating the system’s functional blocks and its high granularity increase the flexibility of the platform for testing reconstruction algorithms;
3. The new platform, together with the finite element method, enables an effective evaluation of RTI reconstruction algorithms.

In terms of creating the sensitivity matrix, the following notes can be made:

1. Narrowing the impact profile between the sensors in the sensitivity matrix increases the quality of image reconstruction (for the LBP method);
2. The inner part of the reconstruction image is characterized by lower values in relation to its boundary zone.

There are also other techniques, such as EIT or ECT [32]. Electrical impedance tomography (EIT) is the closest approach. Measuring the internal conductivity or impedance distribution in a region of interest theoretically allows for the detection of changes induced by a human or other object, but it is usually characterized by lower resolution and sensitivity to environmental fluctuations. The impedance changes can be subtle, requiring the use of very sensitive sensors. Hybrid solutions are currently being developed, but the authors do not delve into this topic in this article. Future research may consider other approaches, including EIT-based methods and more advanced reconstruction algorithms such as those based on deep learning techniques. Moreover, the authors have not yet conducted a detailed analysis of the use of GPUs or FPGAs to accelerate the reconstruction process, but they are aware of the potential benefits of these technologies, especially in the context of real-time applications [33].

The system developed in the current work is not the final solution. It is necessary to conduct further development work, emphasizing the development of algorithms for identifying objects in the reconstructed RTI image and algorithms for verifying the location and geometric properties of the identified objects in the measurement space. The extension of FEM models and reconstruction algorithms allowing for the simulation of rooms of various shapes is also necessary. In addition, these studies will be enriched with advanced quantitative comparative tests of the quality of images obtained by RTI and other tomographic methods, as well as a comparison to real-world radio wave installation.

## 6. Conclusions

The aim of the research presented in this article was to develop algorithms and numerical models that would be used to analyze and reconstruct images from measurement data in radio tomography. Imaging methods in radio tomography were discussed, taking into account noise models. Algorithms for reconstructing images from radio data, including the linear back projection algorithm, were also presented.

A process of verifying reconstruction algorithms using the FEM and selected programming solutions was proposed. The models were prepared using FreeFEM v4.15 software. The source code of the developed models and their visualization were also presented. The paper ends with the results of the simulations performed with the analysis of WIFI signal propagation in measurement areas for different transmitter locations. The novelty of this paper includes the flexible architecture of the RTI evaluation platform with an automated evaluation process.

Automated Evaluation Process: The proposed platform integrates FEM-based simulations with a modular microservices architecture which allows for a fully automated assessment of RTI reconstruction algorithms. This reduces the need for manual intervention and accelerates the development and optimization of imaging techniques.

Enhanced Flexibility and Scalability: By employing a highly granular system design, the presented method allows for a seamless integration of different reconstruction algorithms, facilitating rapid experimentation and adaptation to various application scenarios, such as real-time monitoring in smart buildings.

Comprehensive Simulation Framework: The use of FEM simulations allows for obtaining detailed information on radio wave propagation and provides a robust tool for evaluating algorithm performance under controlled conditions. Since the finite element method has been used for many years, the main advantage of the conducted research is the integration of the tool with the microservices environment.

The presented research confirms the great potential of the developed method and the need for further research on algorithms for assessing the effectiveness of RTI reconstruction methods. The results of the simulations confirm the legitimacy of using FEM to support the analysis of the effectiveness of radio tomography image reconstruction algorithms.

## Figures and Tables

**Table 1:** Execution time of algorithms for forward and inverse problems for the two executions.

Number of Sensors	16			8		
Object Localization	Left	Center	Right	Left	Center	Right
**First execution**						
**Forward problem [s]**	45.43	48.93	46.23	23.02	25.44	24.12
**Compilation [s]**	0.019	0.013	0.012	0.030	0.022	0.022
**Nb of triangles [nb]**	27,674	25,528	25,768	23,920	28,376	23,756
**LBP 32 × 32 [ms]**	0.7	0.6	0.6	0.2	0.3	0.2
**Second execution**						
**Forward problem [s]**	46.44	49.64	47.99	22.17	24.48	23.59
**Compilation [s]**	0.020	0.022	0.022	0.021	0.012	0.014
**Nb of triangles [nb]**	27,674	25,528	25,768	21,720	24,572	23,756
**LBP 32 × 32 [ms]**	0.6	0.7	0.6	0.3	0.2	0.2

## Data Availability

Data are contained within the article.
